# Supplementary material for: Synergistic Effects of Gibberellic Acid, Forchlorfenuron, Thidiazuron, and Brassinosteroid Combinations on Seedless Berry Development and Quality Enhancement in ‘Shine Muscat’ and ‘Red Muscat of Alexandria’ Grapes
Source: Biology (Basel). 2025 Sep 15;14(9):1270. doi: 10.3390/biology14091270 (PMC12467870; doi:10.3390/biology14091270)
Supplement: Supplementary file 1 [file biology-14-01270-s001.zip › biology-3824536-supplementary.pdf]

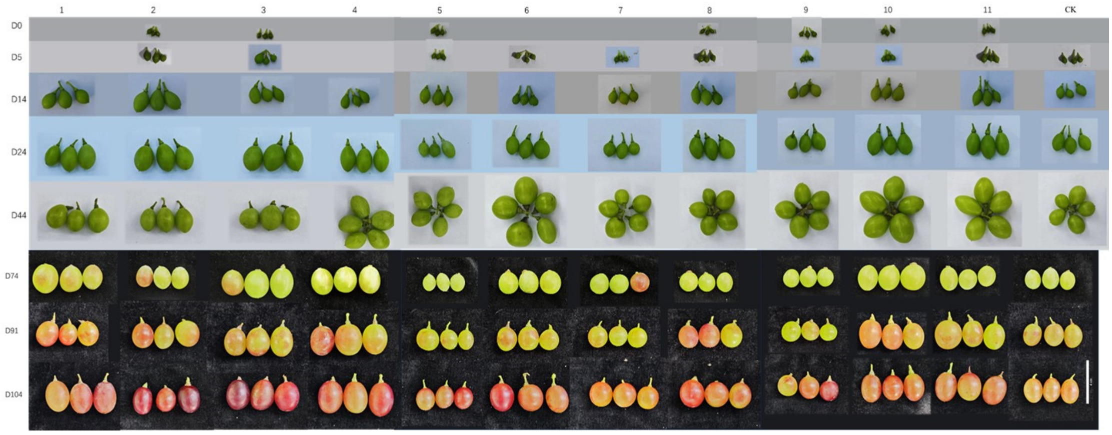

Figure S1. Phenotypic development of 'Red Alexandria' grape berries under various plant - growth regulator (PGR) and streptomycin (SM) treatments. Treatments 1 - 11 correspond to different PGR/SM combinations as detailed in Table 1, with CK representing the water - treated control. The scale bar represents 4 cm."

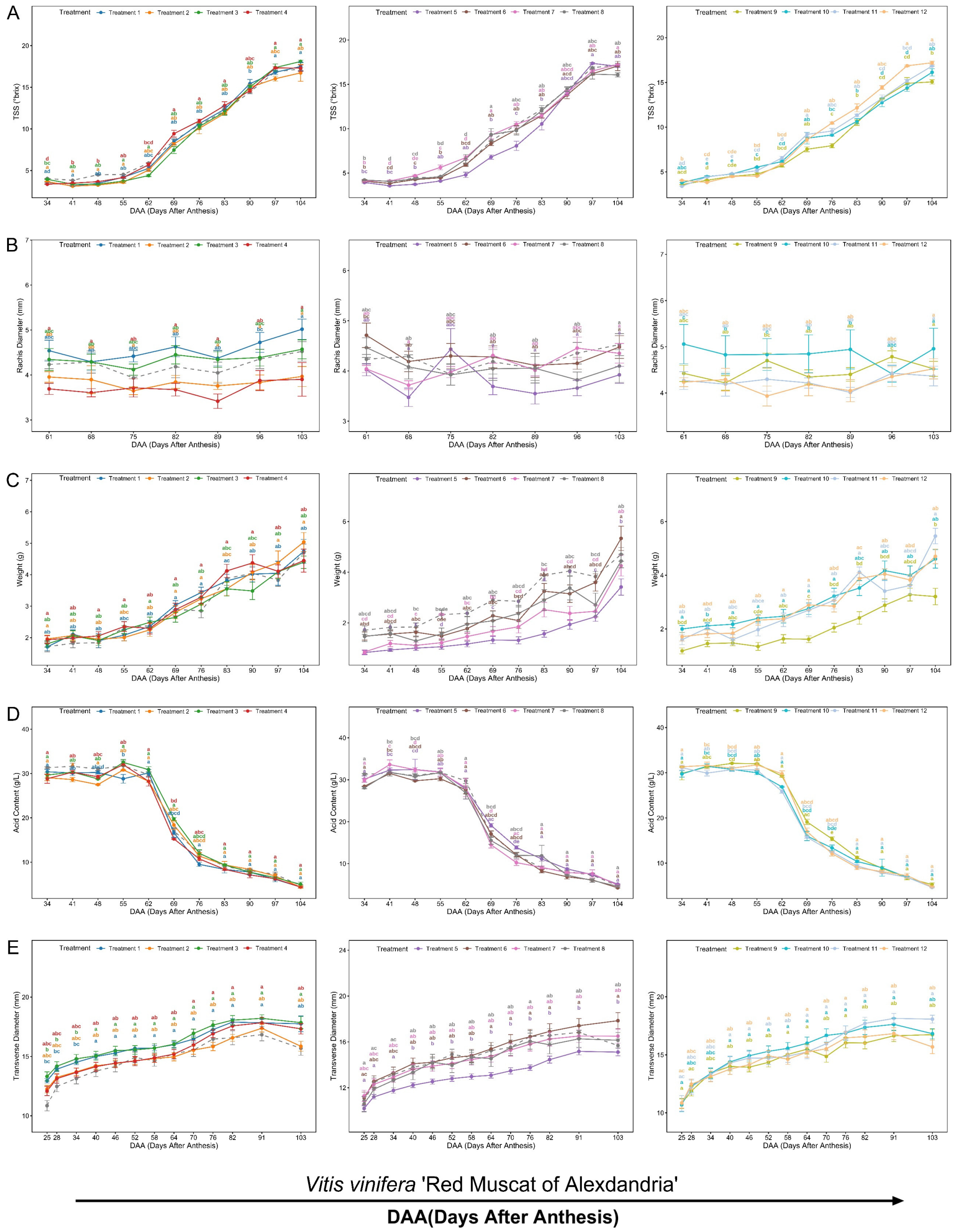

Figure S2.Developmental dynamics of 'Red Muscat of Alexandria' grape berries under plant growth regulator (PGR) and streptomycin (SM) treatments. (A : Total soluble solids (TSS) content. B : Transverse rachis diameter. C : Berry weight. D : Total acidity (TA). E : Transverse berry diameter.Treatment conditions 1–11 correspond to the PGR/SM combinations detailed in Table 1, with CK as the water-treated control. Data are presented as mean values with error bars indicating variability)
